# Urethra-preserving and dorsal capsule fenestration with robot-assisted simple prostatectomy for severe LUTS in small prostate: a case report

**DOI:** 10.3389/fsurg.2024.1497556

**Published:** 2024-11-28

**Authors:** Lijie Wen, Yue Zhang, Yi He, Yang Yu, Bo Yang

**Affiliations:** Department of Urology, The Second Hospital of Dalian Medical University, Dalian, China

**Keywords:** small prostate, benign prostatic hyperplasia, minimally invasive treatment, robotassisted laparoscopy, prostatectomy

## Abstract

**Background:**

Small prostates (volume <30 ml) induce bladder outlet obstruction with pathophysiological changes distinct from those associated with large prostates. Treatment options often include transurethral incision of the prostate (TUIP) or transurethral resection of the prostate (TURP). Existing treatments have issues with high recurrence and complication rates. Therefore, we aim to explore a new minimally invasive surgical approach for patients with severe lower urinary tract symptoms (LUTS) and a small prostate.

**Methods:**

A patient with severe LUTS and a small prostate was admitted to the Department of Urology at the Second Hospital of Dalian Medical University. The patient had no median lobe but presented with multiple bladder stones. Relevant data (IPSS score, urine flow rate, operation time, hemoglobin drop, catheterization time, hospitalization time, residual urine) were collected before and after surgery to assess the safety and efficiency.

**Results:**

The patient was 72 years old with a prostate volume of 22.14 ml, a preoperative IPSS score of 28, PSA of 0.314 ng/ml, maximum urine flow rate of 3.5 ml/s, and a prostate MRI PI-RADS score of 2. The patient underwent robot-assisted cystolithotomy, urethra-preserving prostatectomy and dorsal capsule fenestration. The surgery lasted 105 min, with a postoperative hemoglobin drop of 3 g/L. There was no continuous bladder irrigation postoperatively, and the catheter was removed after 10 days. The patient was hospitalized for 4 days and followed up for 24 months. At 6 months postoperative, the patient had an IPSS score of 6, a QoL score of 1, a urine flow rate of 18 ml/s, and residual urine of 8 ml, with nocturia occurring 1–2 times. At 24 months postoperative, the patient had an IPSS score of 7, a QoL score of 1, a urine flow rate of 21 ml/s, and residual urine of 15 ml, with nocturia occurring 1 time.

**Conclusion:**

Robot-assisted urethra-preserving prostatectomy and dorsal capsule fenestration is a promising alternative treatment for patient with severe LUTS due to a small prostate in both long-term safety and efficacy. Further large-sample controlled studies are needed for additional evaluation and validation.

## 1 Introduction

Normally, a small prostate is defined as having a volume of less than 30 ml (1). According to the European Association of Urology (EAU) 2023 guidelines, transurethral incision of the prostate (TUIP) is recommended to surgically treat moderate-to-severe lower urinary tract symptoms (LUTS) in men with prostate size less than 30 ml, without a middle lobe (2). The bladder neck is incised at 5 and 7 o'clock just distal to both ureteral orifices, and extended down to the verumontanum without removal of the prostate tissues during TUIP (3). TUIP shows comparable efficiency to transurethral resection of the prostate (TURP) in small prostate patients (4). The potential underlying mechanism of TUIP in treating patients with small prostates may involve reducing the pressure exerted on prostatic urethra by bladder neck and prostate fibrosis (5). TUIP is a technique to open the ventral prostatic capsule with two incisions (3). There is a possibility that the incisions may reclose over time, leading to disease recurrence. The Madigan prostatectomy was first described in 1990, with small or fibrous prostates excluded from the study (6). Apart from preservation of an intact unopened urethra, the dorsal prostatic capsule is not going to be closed in Madigan prostatectomy. Considering the potential pathophysiological mechanism of prostate or capsule fibrosis, could the Madigan technique have therapeutic significance for patients with a small prostate and moderate to severe LUTS theoretically? Here, we report a robot-assisted cystolithotomy, urethra-preserving prostatectomy and dorsal capsule fenestration to treat a patient with a small prostate who simultaneously presented with LUTS and bladder stones.

## 2 Case presentation

**Figure 1:**
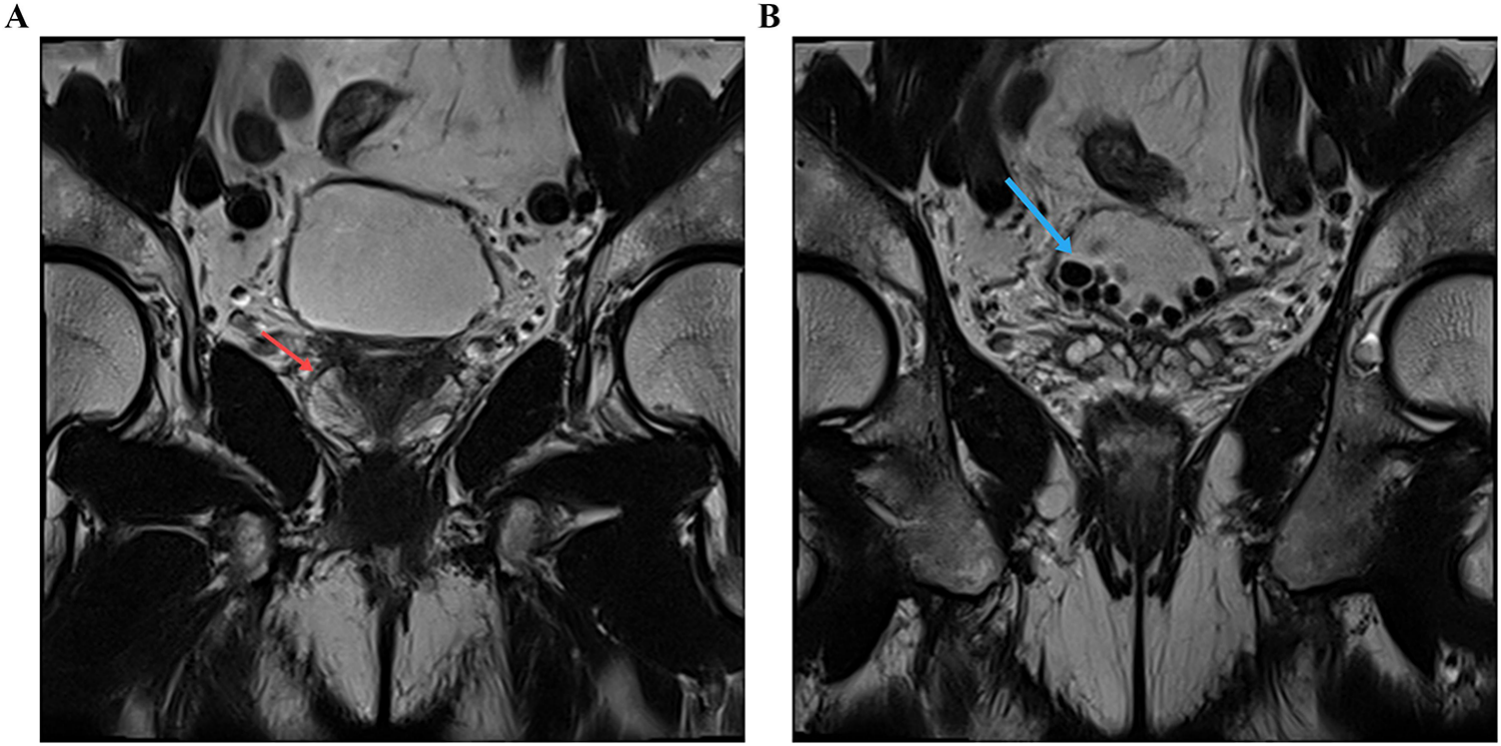
Magnetic resonance imaging revealing the **(A)** enlarged prostate and **(B)** bladder stones.

A 72-year-old man who presented progressive dysuria for 5 years was included. Written informed consent was obtained from the individual for the publication of any potentially identifiable images or data included in this article. The patient is 172 cm tall, weighs 75 kg, and has no past medical history. The International Prostate Symptom Score (IPSS) was 28 and digital rectal examination did not reveal any palpable prostate nodules. Serum prostate specific antigen was 0.314 ng/ml and the prostate volume assessed by MRI was 22.14 ml. MRI examination indicated benign prostatic hyperplasia complicated by multiple bladder stones (Figure 1). Urodynamic test results showed a maximum flow rate of 3.5 ml/s, detrusor pressure at maximum flow rate of 40.3 cmH_2_O, and maximum detrusor pressure of 40.8 cmH_2_O. Over the past 2 years, he has regularly taken tamsulosin and finasteride orally, but his symptoms have not significantly improved. Because he also had multiple bladder stones, after communicating with the patient and obtaining consent, we decided to perform a robot-assisted Madigan prostatectomy and cystolithotomy.

**Figure 2:**
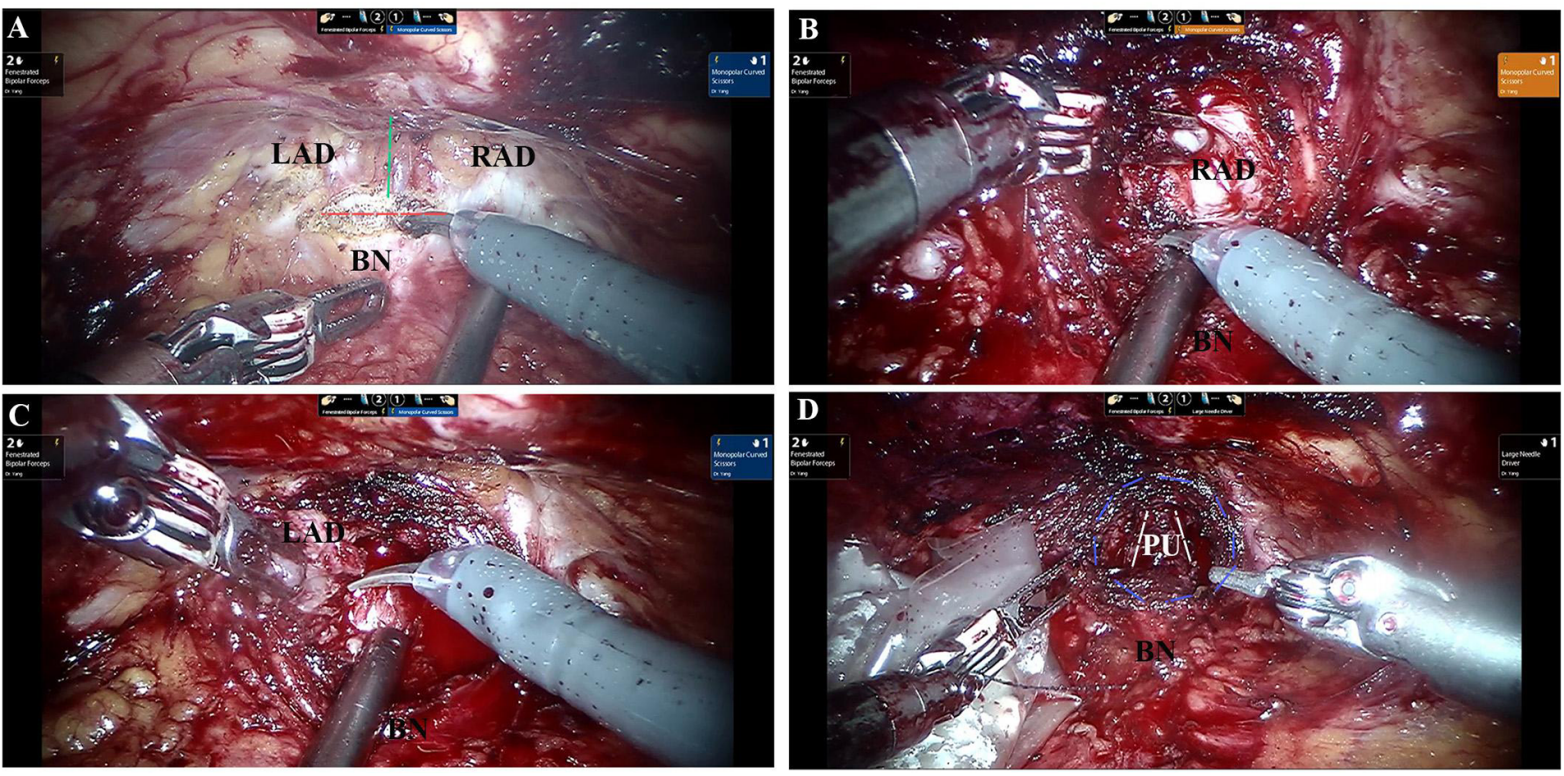
Dissection of the adenoma. **(A)** The horizontal (red line) and longitudinal incision (green line) on the prostate. **(B)** Resection of the right lobe of the prostate. **(C)** Resection of the left lobe of the prostate. **(D)** Appearance of prostatic fossa (purple circle) after removal of adenom with dorsal capsule fenestration. LAD, left adenoma; RAD, right adenoma; BN, bladder neck; PU, prostatic urethra.

Under general anesthesia, the patient was placed in a steep Trendelenburg position. Five-port extraperitoneal approach was used for the prostatectomy with a da Vinci® Si Surgical System (Intuitive Surgical, Sunnyvale, CA, USA) (7). Preprostatic fat was removed after dissecting the retzius space. The junction of bladder and prostate was identified by pulling the catheter. A horizontal incision as long as crossing the base of the prostate was chosen near the distal end of bladder-prostate junction. A longitudinal incision on capsule was deepened by monopolar coagulation until the prostate adenoma was identified (Figure 2A). A longitudinal incision was made at the isthmus of the prostate to divide it into left and right lobes. The urethra was carefully protected when removing the left and right lobes (Figures 2B,C). After the specimens were taken out, the wound was examined and performed strict hemostasis. The dorsal capsule was not closed (Figure 2D). Then we reselected a longitudinal incision on the bladder and completed the cystolithotomy.

Total operating time was 105 min. According to the color of urine after surgery, we gave him no bladder irrigation. By the fourth day after surgery, the patient had resumed a normal diet, and the surgical incision was healing well with no obvious signs of infection. The patient was then discharged as planned. Based on our past experience, for patients with a small prostate, the catheter is typically kept for about 2 weeks post-surgery. For this patient, the catheter was removed on the 10th day after surgery. Postoperatively, follow-up was conducted for 2 years, including IPSS score, urinary flow rate, and residual urine. The follow-up result is shown in Table 1.

**Table 1:** Results of follow-up on IPSS, QoL, average flow rate, and residual volume.

Parameters	6 months postoperative	24 months postoperative
IPSS score	6	7
QoL	1	1
Average flow rate (ml/s)	18	21
Residual volume (ml)	8	15

IPSS, International prostate symptom score; QoL, quality of life.

## 3 Discussion

Voiding symptoms in aging men are typically attributed to bladder outlet obstruction (BOO) resulting from benign prostatic enlargement (8). The enlarged prostate increases urethral resistance, resulting in compensatory changes in bladder function. Obstruction-induced changes of bladder detrusor function lead to urinary frequency, urgency, and nocturia, together with dysuria, which is called LUTS. However, the severity of LUTS does not necessarily correlate with the size of the prostate (9). Previous study showed that BOO patients with a small prostate had significantly higher post-void residual and detrusor underactivity positive rate, which means there could be different pathophysiology from the moderate and large prostate groups (1). Other factors, such as the prostate urethral angle, the prostatic capsule, and the smooth muscle tone in the prostatic stroma, rather than the actual prostate size, have been shown to be related with the pathophysiology in small prostate (10, 11).

Over a period of 4 years, clinical progression of benign prostate hyperplasia (BPH) was observed in 4.6% of men treated with combination medical therapy and 13.2% of men given a placebo (12). This suggests that while medications targeting androgen receptor activity or smooth muscle contraction are generally effective, they do not address all mechanisms contributing to LUTS. There is a higher risk of bladder neck contracture (BNC) after TURP for small prostates, which further confirms the possible pathophysiology of prostatic fibrosis (5). Recently, a systematic review reported that the reoperation rate of TUIP at 5 years was 13.4% (13). TUIP is a technique to open the ventral prostatic capsule with two incisions. There is a possibility that the incisions may reclose over time, leading to disease recurrence. In cases of small prostates, obstruction is caused not only by static compression of the urethra but also by the elastic tension of the bladder neck or prostate due to fibrosis. After resection of the gland in a small prostate during TURP, the space gained in the prostatic urethra may be reduced by the elastic recoil of the peripheral zone, unlike larger prostates where more space is created. Therefore, the mechanism of TUIP is to release tension by incising the peripheral zone. However, transurethral surgery can damage the urethral mucosa, potentially increasing urinary irritation in the short term and leading to scarring in the long term. The Madigan procedure, by preserving the urethral mucosa while removing the peripheral zone, decompresses the bladder neck completely, which has a theoretical advantage in solving the pathogenesis of a small prostate. Compared with the conventional transurethral techniques, Madigan prostatectomy also has other advantages. Xie et al. reported that the patients received Madigan technique had significantly less complication rate of retrograde ejaculation than that in bipolar transurethral resection of the prostate (0 vs. 80%) (14). The drawback is that this procedure is more expensive and invasive than TUIP. The limitation of this study lies in its nature as a single case report, representing an attempt to apply a previous surgical technique to a new indication. Further validation through large-scale randomized controlled trials is needed in the future.

## 4 Conclusion

Robot-assisted urethra-preserving prostatectomy and dorsal capsule fenestration is a promising alternative treatment for patient with severe LUTS due to a small prostate in both long-term safety and efficacy. Further large-sample controlled studies are needed for additional evaluation and validation.

## Data Availability

The raw data supporting the conclusions of this article will be made available by the authors, without undue reservation.
